# An integrative review of parent education approaches in sport: Considerations for program planning and evaluation

**DOI:** 10.1111/sms.14620

**Published:** 2024-04-06

**Authors:** Fabrício João Milan, Camilla J. Knight, Lucas Machado de Oliveira, Vitor Ciampolini, Michel Milistetd

**Affiliations:** ^1^ Department of Physical Education Federal University of Santa Catarina Florianópolis Brazil; ^2^ Department of Sport and Exercise Sciences Swansea University Swansea UK; ^3^ Department of Physical Education and Sport University of Adger Kristiansand Norway

**Keywords:** families, intervention, parent involvement, re‐aim, youth sport

## Abstract

In recent years, there has been an increase in the delivery and evaluation of parent education programs within youth sport. Subsequently, some recent reviews of these programs have been conducted. However, one consistent issue across many of the programs and associated review papers is the lack of an appropriate evaluation framework to guide the planning or associated reporting of the outcomes of the interventions. This has limited understanding of the overall impact of sport parenting interventions. Thus, the purposes of the current study were as follows: (a) to identify commonalities in the reporting and evaluation of parent education programs; (b) to identify gaps in the reporting and evaluation of parent education programs; (c) to draw these insights together to provide suggestions regarding how the RE‐AIM could be used to enhance planning and evaluation of evidence‐based programs for parent education in sport. Specifically, utilizing the RE‐AIM framework to provide insights into pertinent evaluation metrics, this integrative review aimed to identify commonalities and gaps in the reporting of parent education programs. The RE‐AIM framework considers the essential elements to assess the external and internal validity of interventions through five dimensions: Reach, Effectiveness, Adoption, Implementation, and Maintenance (*Am J Public Health*. 1999;89(9):1322‐1327). Subsequently, the review aimed to provide suggestions regarding strategies to enhance the planning and evaluation of evidence‐based programs for parent education in sport. Overall, the analysis demonstrated that most studies presented some pertinent evaluation information related to the RE‐AIM framework, such as the number of participants and contacts made, the measures used, and the program level. However, the studies also lacked information on participant exclusion criteria, the method used to select the delivery agent (e.g., parents engaged in the program), and cost measures. Overall, the current study identified various areas where programs could be enhanced, specifically related to reporting procedural elements (e.g., program design, target population, and costs) pertaining to the implementation of parent education programs.

## 1 INTRODUCTION

The involvement of parents in youth sport is influenced by personal, relational, and environmental/sociocultural factors,^1^, ^2^, ^3^ and consequently, parents are not always able to meet their children's needs and/or preferences in this environment.^3^, ^4^ For instance, at the personal level, parents' behavior concerns impact their involvement in their child's sport.^3^ At the relational level, parents' relationship with their child's coach affects their engagement and comments.^5^ Then, at the environmental/sociocultural level, parents' behavior can be influenced by broader sports cultures in which they are immersed.^1^ To manage the constant demands they encounter and provide optimal support to their children, parents often experiment with different approaches based on previous experiences and “trial‐and‐error” learning.^6^ Additionally, parents seek information from different sources and rely on their relationships with their spouse/partner and family members, other parents, or coaches among others.^6^, ^7^ However, the effectiveness and appropriateness of such strategies are not always clear,^8^ and some parents are perceived as inappropriately involved in youth sport.^1^ Recognizing these challenges, researchers have made efforts to support and educate parents to enhance their involvement in sport.^9^

In recent years, particular consideration has been given to identifying the types of information and support from which parents may benefit^3^, ^10^, ^11^ and recommending how education programs can be structured.^12^ For instance, Dorsch et al.^10^ and Thrower et al.^13^ highlighted the need for parents to receive appropriate information about the sports landscape and how they can be positively involved in their children's sports trajectory. Meanwhile, a variety of education programs have been delivered to inform parents about their role and their child's development and also to effectively involve them in the sports system.^11^, ^14^, ^15^

As increasing numbers of interventions are conducted with parents, evaluating these programs is critical.^8^ Specifically, there is a need to identify barriers and facilitators to effective delivery, convey the relevance of such interventions to sports organizations, and identify requirements to promote better interventions in the future. To date, individual interventions have devoted attention to assessing pre‐ and post‐intervention parental behavior change.^15^, ^16^, ^17^, ^18^, ^19^ Further, evaluations have also highlighted a range of challenges associated with interventions, such as program engagement and implementation (participant attrition) or delivery and effectiveness.^14^, ^15^, ^16^, ^18^ Recently, these challenges have been consolidated in a review by Burke and Colleagues.^8^

Despite the usefulness of individual evaluations and the recent review of the interventions, there have been no attempts to synthesize intervention information on evaluation frameworks. Drawing on an evaluation framework is needed because many studies have demonstrated concerns about the validity of the interventions^14^ and the fidelity of the programs offered.^19^ Moreover, given the ongoing issues identified globally regarding parental involvement in youth sport, such as pressure on children, inappropriate comments on the sidelines, and abuse toward referees (see Dorsch et al.^12^ Knight et al.^20^ Webb & Knight^21^ for examples), there is a need to consider broad evaluation elements that are pertinent to sports organizations and identify the elements of interventions that need to be enhanced to support the transferability and feasibility of education programs across sport organizations and stakeholders.^16^ Overall, evaluation frameworks provide a synthesizing architecture to evaluate intervention implementations in specific populations and settings^22^ and their integration into sport parenting interventions has been recognized as an avenue that requires exploration to improve the quality of the programs offered.^8^

Aligned with these recommendations, the purpose of the current study was to review existing sport parenting interventions utilizing an appropriate evaluation framework. The RE‐AIM framework was identified as an appropriate theoretical scaffold against which to evaluate parent education programs because it considers the essential elements to assess the external validity (e.g., generalizability and long‐term change) and internal validity (e.g., impact) of interventions. The RE‐AIM was originally developed in the field of health promotion to evaluate public health interventions. The focus was on advancing aspects of intervention settings drawing upon social‐ecological perspectives. This occurs through a coding sheet containing 21 items^23^ divided into five dimensions: Reach, Effectiveness, Adoption, Implementation, and Maintenance^24^ (see https://re‐aim.org/learn/).

Recently, Thrower et al.^25^ evaluated a tennis parent education workshop through the RE‐AIM lens. This study provided an important illustration of how RE‐AIM can be used to inform the planning and evaluation of sport parent education programs. Particularly, by drawing on RE‐AIM, Thrower and colleagues were able to examine the essential characteristics of their program and identify the varied areas of success and limitations. By using such an approach to conduct a collective evaluation of programs conducted to‐date, there is an opportunity to expand our understanding of both individual‐ (i.e., parent experience) and organization‐level impact (i.e., cultural changes in sport organization) of the programs^14^, ^16^, ^19^ and clearly identify areas that have been neglected or undervalued in evaluations to‐date.

It is important to recognize that these programs were not explicitly developed using the RE‐AIM framework, and so the purpose of the current study *is not* to judge the quality of the studies regarding their alignment with RE‐AIM. Rather, as the field of sport parenting has progressed from descriptive to intricate/multifaceted examinations of parents' experiences in sports,^12^ it is hoped this study will facilitate a positive effort to enhance the discussion of holistic approaches to sport parenting interventions and evaluations.^25^ Specifically, as a well‐utilized and established theoretical framework, the purposes of the current study were as follows: (a) to identify commonalities in the reporting and evaluation of parent education programs; (b) to identify gaps in the reporting and evaluation of parent education programs; (c) to draw these insights together to provide suggestions regarding how the RE‐AIM could be used to enhance planning and evaluation of evidence‐based programs for parent education in sport.

## 2 METHOD

**FIGURE 1:**
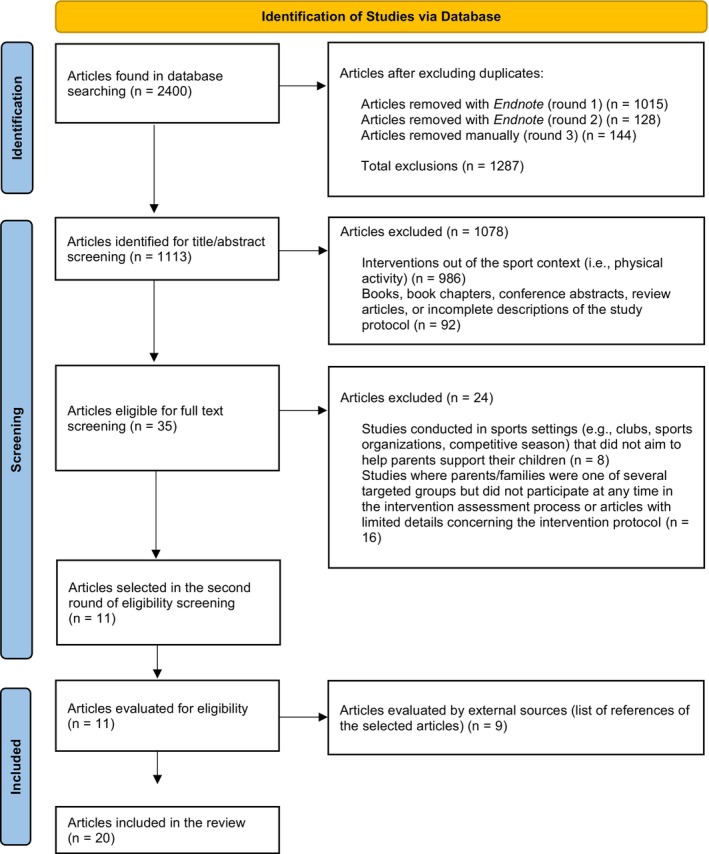
Identification of studies via database.

Integrative reviews are valuable tools for consolidating a field of study or a specific discipline and uncovering key aspects that affect the investigated phenomenon.^26^ Although an integrative review does not always involve quantifying a specific number of articles from the field or discipline, it can reveal some generalizations and potential commonalities/issues. As a result, the insights gained through an integrative review can help better understand and connect related work areas on a particular topic of interest.^27^ Once our study's objective and scope were defined, the review process was guided by the Preferred Reporting Items for Systematic Reviews and Meta‐Analysis (PRISMA) checklist,^28^ detailed in Figure 1. The RE‐AIM framework (through the RE‐AIM coding sheet) was used to guide analysis.^24^

### 2.1 Identification

To address the study's purpose, original articles on sport parent education were searched through keyword and abstract search combining terms and Boolean operators [for example, (parent* education OR parent* training) AND (program OR online program* OR web‐based) AND (youth sport)]. English peer‐reviewed manuscripts were acquired across multiple electronic databases, including Web of Knowledge, Scopus, ERIC, PubMed, SPORTDiscus, and PsycINFO, between 2001 and 2022*—this interval represents the contemporary period of parental involvement literature, where educational strategies to support parents became explicit in sport.^12^

### 2.2 Screening and inclusion

Screening and inclusion of articles consisted of three steps. First, the lead and the third author, who had 3 and 1 year of experience conducting reviews, respectively, individually screened studies by title and abstract reading (e.g., unrelated and duplicated articles were removed). For inclusion, the articles were required to provide descriptions or evaluations of programs with parents/families (i.e., a program conducted with parents or families aimed to improve knowledge and specific support to parents in sports)† (Table 1). A third experienced researcher with over 5 years of expertise in the sports science field—the fifth author in this paper—assisted in the final decision in the few instances where a disagreement occurred.

**TABLE 1:** Inclusion and exclusion criteria for articles.

Component	Inclusion criteria	Exclusion criteria
Applied throughout the search process (i.e., identification and screening)		
Date range	January 2000 to December 2022	–
Language	English‐language journals only	–
Intervention type	Descriptions or evaluations of intervention with parents/families (i.e., an intervention conducted with parents or families, and which aims to improve knowledge and specific support to parents in sports)	Interventions out of the sport context (i.e., physical activity)
Study type	Original quantitative and qualitative research (with protocol description and evaluation process)	Book, book chapter, conference abstract, position paper, or another type of publication that was not an original article
Applied to assess the eligibility		
Focus of intervention	–	Studies conducted in sports settings (e.g., clubs, sports organizations, and competitive season) that did not aim to help parents support their children
Level of intervention	–	Studies where parents/families were one of several targeted groups but did not participate at any time in the intervention assessment process or articles with limited details concerning the intervention protocol

*Note*: Inclusion criteria were applied throughout the search process (i.e., identification and screening), whereas exclusion criteria were applied to assess the eligibility of the 35 articles identified as sports parent education interventions.

Secondly, the first and third authors conducted a full‐text screen. In this process, the exclusion criteria regarding the eligibility assessment of the articles were applied (Table 1). To ensure all relevant studies were identified, the third step was a manual search for articles by screening the references of those selected, which revealed nine articles that met the inclusion criteria for eligibility and were part of the final set of articles for coding. At the end of the screening and inclusion process, 20 articles were identified. From these papers, specific design aspects that had been used to create and deliver parent education programs using the RE‐AIM coding sheet were thematically interpreted.

### 2.3 Coding protocol

Before coding the articles selected for this review, the first and third authors were intimately involved in a training process with the last author to comprehend the checklist indicators for assessment. Consequently, both independently coded one study to assess inter and intra‐rater reliability (the first author had previous experience conducting a full integrative review, while the third author had no experience in that field). After answering yes/no questions and determining explanations about indicators, the coder team discussion was focused on reflecting on the thinking that lay behind screening decisions, especially those that diverged from the “correct” decision. Inter‐coder agreement and reliability were assessed using the Kappa index for intra‐ and inter‐rater measures.^29^ Intra‐rater reliability was calculated by comparing coding on a single article performed at two different and distant times (e.g., later coding 2 weeks after the initial coding). The agreement of the raters was considered consistent between the first author (*Κ* = 0.88, ±0.05) and the third author (*Κ* = 0.79, ±0.05). Inter‐rater reliability was calculated by comparing the coding of the same article by two raters separately, with excellent agreement between 81% and 100% (*Κ* = 0.85, ±0.05).

### 2.4 RE‐AIM coding sheet and adaptation

The RE‐AIM coding sheet is a tool that provides researchers and practitioners with a detailed explanation of the steps involved in conducting a program. The original RE‐AIM coding sheet comprises 21 items as indicators for each of the five dimensions (see http://www.re‐aim.org/). Examples of items include the method to identify the target population, the level of expertise of the delivery agent, and the cost measures. As the RE‐AIM was structured to evaluate programs in public health,^30^, ^31^ specific changes were made to the coding sheet to better align with parent education programs in sport. As Evans et al.'s^30^ study was also conducted in the sport context, this was used as a starting point for the adaptations in the current study. Following adaptation, the final coding sheet used in this study comprised 35 items (20 items from the original version and 15 new; See Data S1).

#### 2.4.1 Reach

This dimension is composed of nine items (five of them referring to the original version of the RE‐AIM and four additional ones) that allowed the identification of information related to the general descriptive characteristics of the population involved in the program (e.g., sample size and participation rate, and description of the target population).

#### 2.4.2 Effectiveness

This dimension is composed of seven items (three related to the original and four additional) that captured information related to the effectiveness of the program, potential behavior changes in the participants, and the extent to which participants engaged in the program (e.g., measures/results, and were the parents/family members who withdrew from the program evaluated or only those who were hired).

#### 2.4.3 Adoption

This dimension is composed of eight items (six from the original and two additional) that allowed a better understanding of the program context, the credentials of the individual who delivered the program, and the organization's characteristics (e.g., description of the program location and delivery agent expertise level).

#### 2.4.4 Implementation

This dimension comprises seven items (three from the original and four additional) that enabled the identification of the program delivery design and theoretical support for the program (e.g., number of program contacts, cost measures, and pilot test).

#### 2.4.5 Maintenance

This dimension is composed of four items (three from the original and one additional) that provide information on the program evaluation, outreach (e.g., regional, national, and international), and modifications needed (e.g., has the program has been modified and at what level of program was the program in place).

The RE‐AIM coding sheet was used to interpretatively assess the design of parent education programs in sports. Throughout the items, “yes” was noted for present indicators and “no” for absent ones. The percentage of items present or missing were then calculated.

## 3 RESULTS AND DISCUSSION

### 3.1 The RE‐AIM perspective: Identifying similarities and differences in the reporting and evaluation of parent education programs

Across the 20 studies (Table 2), all scored at least one indicator in the Effectiveness, Implementation, and Maintenance dimensions. However, two indicators received a score of zero on at least one study—one for reach and one for adoption (Table 3).

**TABLE 2:** Characterization of the studies selected.

Author (year)	Research Design	Participants	Country	Sport	Intervention delivery method
Harwood and Swain^41^	Mixed Methods	16 parents, youth athletes, and coaches	United Kingdom	Tennis	90‐min face‐to‐face sessions; self‐directed tasks; booklet
Smoll, Smith and Cumming^34^	Quantitative	327 parents, youth athletes, and coaches	United States	Basketball	75‐min face‐to‐face workshops; 28‐page booklet
Bapat, Jorm and Lawrence^35^	Quantitative	40 parents, senior/youth athletes, and coaches	Australia	Football and Netball	8‐h training sessions; youth mental health first aid manual
Richards and Winter^33^	Mixed Methods	21 parents	United Kingdom	Gymnastic	20‐ to 30‐min educational sessions; folder with program orientation
Vincent and Christensen^42^	Qualitative	Parents, coaches, and program directors^a^	United States	Soccer	1‐h face‐to‐face workshops
Glang et al.^36^	Mixed Methods	1004 parents, 4804 youth athletes, and 25 school administrators	United States	High School	Web‐based program (training and resources)
MacDonald and Hauber^39^	Quantitative	47 parents	United States	Contact Sports	30‐ to 40‐min online program; information handout; youtube video
Dorsch et al.^14^	Quantitative	162 parents and youth athletes	United States	Soccer	33‐page Sport Parent Guide; 45‐minute Sport Parent Seminar
Thrower, Harwood and Spray^11^	Qualitative	31 parents	United Kingdom	Tennis	Six face‐to‐face workshops
Hurley et al.^32^	Mixed Methods	66 parents	Australia	Rugby	1‐h face‐to‐face workshop (supplemented by print and online content)
Azimi and Tamminen^40^	Mixed Methods	20 parents and youth athletes	Canada	Hockey	45‐min workshop; parent handbook
Lisinskiene and Lochbaum^17^	Qualitative	20 parents and youth athletes	Lithuania	Martial Arts	12‐month face‐to‐face program (consultation, 1‐h theory class, and 60‐min training class)
Rice and Curtis^18^	Quantitative	140 parents	United States	Multiple Sports^b^	Online video and print educational intervention (educational fact sheets, booklets, wallet cards, clipboard stickers, and videos)
Sampol et al.^38^	Quantitative	12 football teams^a^	Spain	Football	Face‐to‐face socio‐educational intervention
Thrower, Harwood and Spray^15^	Mixed Methods	38 parents	United Kingdom	Tennis	Online education program (eight short online videos, quizzes, supplementary downloadable materials)
Hurley et al.^16^	Qualitative	17 parents	Australia	Rugby	1‐h face‐to‐face workshop (supplemented by print and online content)
Kwon, Elliott and Velardo^43^	Qualitative	21 parents and coaches	Australia	Soccer	Three short videos (ranging from 45 to 90 s)
Tamminen, et al.^19^	Quantitative	366 athletes	Canada	Hockey	1 h of a series of online video modules (supplementary downloadable/printable materials)
Hurley et al.^37^	Quantitative	540 parents	Australia	Football, Australian Rules Football and Cricket	50‐ to 75‐min face‐to‐face workshops (pamphlet and other online resources)
Thrower, Spray and Harwood^25^	Mixed Methods	130 parents and 130 children	United Kingdom	Tennis	2‐h face‐to‐face workshop (two online versions); 8‐page pre‐workshop booklet; free online webinars (due to the COVID‐19 pandemic)

^a^ Note: participant number is unclear.

^b^ Examples of sports include football, soccer, lacrosse, basketball, hockey, wrestling, baseball, volleyball, softball, cheerleading, gymnastics, swimming, flag football, tennis, equestrian, cross country, archery/air rifle, and dance.

**TABLE 3:** RE‐AIM dimensions (20 accountable items) analyzed in the studies (*n* = 20).

Dimension	Reported Items	Percentage
	*n*.	%
Reach		
Method to identify the target population	16	80
Inclusion criteria	14	70
Exclusion criteria (individuals ineligible)	0	0
Sample size and participation rate	19	95
Characteristics of participation and non‐participation	5	25
Reach dimension average	10.8	54
Effectiveness		
Measures/results (in the shortest assessment)	20	100
Were the parents/family members who withdrew from the intervention evaluated or only those who were hired?	15	75
Attrition percentage (parents/family members completing the program)	11	55
Effectiveness dimension average	15.3	76.6
Adoption		
Description of the intervention location	7	35
Description of the team that delivered the intervention	12	60
Method to identify the target delivery agent	0	0
Delivery agent expertise level	8	40
Inclusion/exclusion criteria (team delivered intervention\organizations)	9	45
Adoption rate (#participating configurations/total configurations)	11	55
Adoption dimension average	7.8	39.1
Implementation		
Number of intervention contacts	20	100
Extension protocol delivered as intended	17	85
Cost measures	1	5
Implementation dimension average	12.6	63.3
Maintenance		
Was individual behavior assessed at any time after completion of the intervention?	18	90
At what level of intervention was the program in place?	19	95
Has the program been modified? (suggestions)	10	50
Maintenance dimension average	15.6	78.3

### 3.2 Reach

The reach dimension refers to representativeness and the ways through which an individual can participate in a program. Approximately half of the indicators suggested by the RE‐AIM framework were included in the studies (see Table 3). The target population was identified through two approaches. First, either the program (and by extension the population to target) was requested by a sports organizations or clubs as they perceived it would be beneficial for their parents.^17^, ^32^, ^33^, ^34^ Second, the research team independently identified parents to whom they had access and could deliver their education program. For instance, the program appeared to be motivated by a pre‐existing relationship between the researcher and the club/organization.^11^, ^15^, ^25^

Most studies (17 studies) described the pertinent demographics of those involved with the program. In 11 studies, programs have been predominantly attended/completed by mothers.^11^, ^14^, ^15^, ^16^, ^18^, ^25^, ^32^, ^33^, ^35^, ^36^, ^37^ Meanwhile, there was an example of reporting only the sports organizations.^38^ There was a concern about reporting the age of the children/athletes corresponding to the parents participating in the program, which ranged from 5 to 19 years (especially between 13 and 15 years). Providing the age of children/athletes could be a pertinent indicator to develop appropriate program strategies for parents and their children. Nevertheless, detailed demographic information is missing (e.g., characteristics of participation and non‐participation, denominator target population number, and what criteria made parents ineligible to participate).

Studies detailed different criteria for inclusion to determine who was eligible to participate in the program. Examples of inclusion criteria were being a parent involved in sports activities, assisting their child in sports, being part of the selected sports organization, and being able to participate.^14^, ^17^, ^36^, ^39^ When athletes were participants, they had to be enrolled in a sports organization (training or competing), have parents attending their sports, and answer pre‐season questionnaires.^34^, ^40^, ^41^ As a result, parents and children engaged in the programs in various ways. In 13 studies, only parents participated (e.g., Hurley et al.,^37^ Sampol et al.,^38^ Thrower et al.,^11^, ^15^ and Thrower et al.^25^); in two studies, both parents and children participated,^32^, ^42^ and in one study, just children (athletes) participated.^19^ There were two programs in which parents, children, and coaches participated,^34^, ^41^ as well as parents and coaches/administrators.^35^, ^43^ It should be noted that some studies did not clarify the participants' eligibility or provided little detail on the inclusion criteria used.^18^, ^19^, ^33^, ^35^, ^38^, ^42^

Regarding reach, based on the reviewed studies, there is a need to report individual‐level outcomes, which means expanding participant and study demographic information to provide a complete picture of who is engaging with the program.^23^ Although the number of participants who took part in programs was mentioned, the percentage of the possible participants this accounted for was largely missing. This prevents a full understanding of engagement with programs and, as Kessler et al.^44^ explained, simply reporting the number of participants does not represent a valid denominator for the program. For example, in Thrower et al.'s^15^ program, the denominator used was the number of parents (62 parents from 30 different tennis centers) who registered interest in participating through an invitation from the sports organization. Even though this information provides some appraisal of the context, it does not allow further reflections on how many parents were potential participants at each tennis center. Understandably, such information may be difficult to provide accurately, but a rough estimate based on the number of children registered at a club or with an organization may provide some indication. An illustration can be found in Thrower et al.'s^25^ research, where they stated that around 7500 parents could potentially participate—data revealed that 13.91% of parents showed an interest in joining the program. Including this information in program evaluations may help support understanding if certain programs, delivery methods, or content are more or less appealing to our potential audiences.

Regarding non‐participation characteristics, this was very difficult to obtain (five studies^17^, ^19^, ^32^, ^35^, ^43^) or did not apply in most of the studies. In general, as highlighted by Lisinskiene and Lochbaum,^17^ within their study parents were highly motivated to join the program because they knew it was for the benefit of their child. However, to advance our understanding of the program's reach, seeking non‐participant data would be very beneficial. This is particularly important to ensure that programs are not only appealing to or impacting parents from specific backgrounds, experiences, or profiles^43^ or that program structures fit parents' needs. Using different methods to reach parents is essential, and Lawrason et al's.^45^ suggestion of applying the RE‐AIM and dividing the outreach strategies into indirect, intended, and direct may be helpful. Additionally, we would also encourage researchers to provide insights into exclusion criteria. This means researchers need to elucidate what makes parents ineligible to participate in the program—instead of exclusion criteria for research. Such improvement would directly influence which parent populations the programs are reaching.^46^

### 3.3 Effectiveness

Most of the aspects recommended in the RE‐AIM effectiveness dimension were addressed (see Table 3). The use of specific questionnaires to measure the impact of the program (e.g., The Sport Emotion Questionnaire—SEQ, Jones et al.^47^; The Sport Anxiety Scale‐2—SAS‐2, Smith et al.^48^; The Mental Health Literacy Scale, O'Connor & Casey^49^) was dominant as a quantitative measure. In general, the questionnaires targeted children,^14^, ^19^, ^34^, ^40^, ^41^ parents,^15^, ^18^, ^32^, ^33^, ^37^, ^39^ or both.^25^, ^35^, ^36^ In order to assess the impact of grassroots football programs on parental attitudes, Sampol et al.^38^ utilized an observational tool called the Parents' Observation Instrument at Sport Events (POISE).^50^ Studies that adopted a qualitative approach used interviews, social validation feedback, diaries (video and reflective), and focus groups. Parents were the predominant participants in 18 studies (e.g., Azimi & Tamminen,^40^ Harwood & Swain,^41^ Hurley et al.,^32^ Hurley et al.,^16^ Kwon et al.,^43^ Lisinskiene & Lochbaum,^17^ Richards & Winter,^33^ Thrower et al.,^11^, ^15^ Vincent & Christensen^42^). However, coaches,^43^ athletes,^40^ and administrators^36^, ^42^ were also included.

In general, when evaluating program effectiveness, the studies collected parents' perceptions only for those who completed the program. There were a few instances (three studies) in which those who did not complete the program were also included in the evaluation.^11^, ^15^, ^32^ Except for three studies in which information regarding the effective participation of parents was unclear,^35^, ^38^, ^42^ the participation and completion rates of the programs were commonly detailed as a demonstration of program effectiveness.

Although five studies did not clarify the barriers or challenges to program effectiveness,^11^, ^14^, ^35^, ^38^, ^41^ some aspects were detailed, such as recruitment of parents, monitoring participation and completion rates, the limited engagement of parents, clarifying instructions for some contents of the program, communicating and establishing rapport with participants, and convincing parents of the importance of the program.^15^, ^16^, ^25^, ^32^, ^33^, ^39^, ^42^, ^43^

As detailed and aligned with research in other settings,^30^, ^31^, ^44^, ^51^ 15 studies provided excellent insights into the effectiveness of their programs, such as clarifying if withdrew parents were evaluated in the program or only those who have participated in the whole implementation. However, researchers must recognize the value of expanding their focus beyond just program effectiveness. Such a concern has been raised by others,^30^, ^44^, ^51^ highlighting that focusing only on program effectiveness can come at the detriment of an interactive process, where more priority is given to effectiveness (in numbers) and less to planning and evaluating with a focus on the feasibility and/or sustainability of the program.^31^, ^51^ Although the program's data indicated notable changes in how children perceive their parents' behavior, Thrower et al.^25^ found that this was mainly reported by parents of athletes under the age of 11. Through the RE‐AIM insights, these findings highlighted a crucial link between the effectiveness and reach of programs, emphasizing the importance of adopting more proactive recruitment strategies and targeting parents, especially those with adolescents, through direct approaches (such as via coaches).^25^

Nevertheless, although the effectiveness dimension had the highest average score (see Table 3), some elements would still benefit from further consideration in future programs. For example, attrition measures related to follow‐up responses (i.e., the ratio of parents who started and parents who did not complete the program) would be useful to provide insights into which programs are more effective at keeping parents engaged (cf. Shelton et al.^46^) and any aspects that may lead to dropout (i.e., the irrelevance of the content and lack of time to participate; Thrower et al.^15^). Among the studies that demonstrated dropout data, Hurley et al.,^32^ Thrower et al.,^11^, ^15^ and Thrower et al.^25^ dedicated strategies to listening to parents who did not complete the program (using questionnaires, participant reflective diaries, forms for feedback after each workshop, comments and forum posts on online programs, and e‐mails), demonstrating that gaining such information is possible. With such insights, researchers and practitioners will be better positioned to understand cultural and contextual changes that may be needed for program sites and members if they were to transfer to other settings.^46^

### 3.4 Adoption

The information covered within the adoption dimension focuses on describing the program (i.e., location, inclusion/exclusion criteria to adopt the program, and adoption rate), and the team responsible for delivering the program. This dimension does not appear to have been considered in many studies (due to the less average items reported; see Table 3). Concerning where the program took place, Tamminen et al.^19^ extensively described the location of the program; for instance, highlighting information regarding the RiSPP (Respect in Sport Parent Program); the context of the study (i.e., Ontario Minor (Ice) Hockey Association), the mandatory nature of the program, the number of youth participating in the league, etc. Similarly, Kwon et al^43^ explained their intervention was explicitly delivered in a particular area in South Australia, with a targeted program for parents whose children were part of soccer clubs in areas of low‐socio‐economic status.

When present (nine studies), the inclusion/exclusion criteria for selecting the participating organizations were attributed to socio‐economic indices (e.g., those from some socio‐economic regions) and prior involvement of the researchers with the organization (e.g., development of previous projects in the sports club). Specifically, most studies (but one) had no data regarding whether some organizations were eligible to participate in their programs besides the ones that did take part. The one exception is Kwon's et al.^43^ study, which indicated that 76 organizations were eligible/contacted, and four participated.‡

Within the studies, it was sometimes difficult to identify the people responsible for developing and delivering the program (four studies did not clarify this data) or how they were selected (no study applied a method to identify the delivery agent, only described them). It appears that in most cases, delivery was conducted by the authors of the studies (12 studies), some of whom are also applied practitioners (i.e., coach educators or sport psychologists), but many of whom work solely in a research capacity. In six studies, it seems that other individuals were involved in the program design and/or delivery and that these individuals were experts in specific professional areas, such as mental health clinician trainers, sports psychologists, representatives from local junior sporting clubs, and health care providers.^17^, ^18^, ^19^, ^25^, ^35^, ^36^ Additionally, we should note that Thrower et al.^25^ delivered the program—study authors—and selected 20 tutors with prior experience in sport and exercise psychology to assist in the program implementation. The tutors underwent a 3‐h training session conducted by the research team.

Aligned with Evans et al.'s^30^ evaluation of coaching programs, much detail was lacking in the parent education program papers pertaining to adoption, which led to the lowest average score among the five dimensions of the RE‐AIM framework (see Table 3). A lack of attention to describing the delivery team, their level of expertise, and the methods for identifying those responsible for delivery reveals implications for the entire program.^24^

First, at the setting level, fully describing the adoption dimension helps us determine the best path for the organization and the program being structured.^16^, ^45^ In this case, researchers and practitioners are unquestionable partners in developing and delivering the program, if they work together with the organization's decision‐makers. In addition, it is important to pay attention to the program context. This means that defining a delivery team could depend, for example, on whether the program for parents is a policy change or an environmental change program.^23^ In the study conducted by Thrower et al.,^25^ a contractual knowledge exchange partnership was established between the sport organization, the first and last authors, and their academic institutions. This partnership was crucial in the development, implementation, and assessment of the program. The research team considered the sport organization's key performance indicators and determined the ideal number of workshops and participants to be reached across England, Scotland, and Wales.

Another important issue in the adoption dimension is the description of the program site, directly aligned with delivery team decisions. When describing the site of the program, it is necessary for programs with parents to seek to understand the current priorities, mission, and/or external factors that may justify the organization's decision to participate or not participate and subsequently provide insight into the adoption rates.^52^ To gain such insights, it is likely that researchers will need to spend considerable time with the organization and have a genuine desire to understand what is likely to work with them and fit their mission. This is time‐consuming, but it is likely that with such insights and ideally working in a truly collaborative manner through co‐producing the program, the potential of buy‐in from organizations and, subsequently, clubs, coaches, and parents within these organizations may increase. Drawing on methods such as action research may also be beneficial as such approaches focus specifically upon evaluating the gaps and needs of a setting and responding to these.^3^

### 3.5 Implementation

Although the studies provided several details regarding the program's implementation, aspects of this dimension may still benefit from further consideration and inclusion (see Table 3). Overall, 17 studies presented in detail the program protocol followed (e.g., theoretical support, program contacts, and pilot test). Furthermore, robust theoretical approaches, specialized models, frameworks, or guides in areas such as mental health were used to support the purposes of the implementation.^14^, ^16^, ^32^, ^35^, ^36^, ^37^, ^39^, ^40^

Delivery occurred through workshop sessions consisting of videos, quizzes, forums, guidance through parenting manuals/guides, presentations, group exercises, brainstorming, audio diaries, webinars, and homework assignments. The structure of the programs corresponded to a single session/module or a set of these (up to a maximum of seven sessions/modules in a single program) that were delivered, ranging from 3 to 12 weeks. The program of Lisinskiene and Lochbaum^17^ was an exception by lasting 1 year through monthly 1‐hour meetings with parents and children.§ Meanwhile, Dorsch et al.^14^ also implemented a booklet (The Sport Parent Guide) as a partial‐implementation condition for comparison among two other groups, a full‐implementation condition (attended a 45‐min Sports Parent Seminar and the Sports Parent Guide), and a non‐implementation condition (attended neither the seminar nor the guide). The time of the sessions ranged from 10 to 180 min.

One study indicated the cost of implementing the program (time spent and budget invested). Thrower et al.^25^ found that the program implementation cost in 2018 was about £15 000, including consultancy fees, printing and promotion expenses, and tutor fees. As an important part of the programs' delivery, three programs conducted a pilot study,^11^, ^25^, ^36^ and another two were pilot studies themselves.^14^, ^33^ Pilot studies are essential in providing information about the program's delivery and effectiveness and ensuring that any necessary issues are addressed prior to roll‐out. For instance, they could help identify that the low attendance of parents in the program could be due to ineffective recruitment strategies used before the program's start or that certain delivery approaches are more or less effective with the target population.

Overall, when considering implementation there are similar results to those found by Evans et al.^30^ with regard to coaching programs. Reports about what was accomplished in the program (detailing the delivery protocol, frequency of contacts, and theoretical support) were common, while pilot testing, consistency, possible adaptations, and cost measures of the entire program process (e.g., time and money) were less frequently reported. Consequently, with regard to the programs included within this review, it appears that the most challenging aspect regarding these points is the follow‐up and demonstration of possible adaptations to the programs. The programs by Richards and Winter^33^ and Vincent and Christensen^42^ did briefly highlight some of the adaptations they made to their study. For example, when describing the sessions that were delivered to the parents, the program manager always reminded the parents why they were participating in the program, realizing the need to avoid conflict between them. Furthermore, Thrower et al.^25^ made changes to the workshop format by switching from face‐to‐face sessions to virtual ones (i.e., webinars) due to the COVID‐19 pandemic. As an illustration, changing the workshop format resulted in more parents attending. Conversely, introducing a non‐profit fee to cover program expenses led to a decrease in parent participation. Thus, even considering that programs were implemented as planned, both cases illustrated the relevance of evaluating the compatibility, which means assessing the actual implementation of the program compared to the initial plan.^45^ With regard to the use of RE‐AIM in parent education programs, Holtrop et al.^23^ highlighted that it is very important to consider adaptations to understand what may be needed for future applications. The logic of monitoring adaptations also has a relationship with the maintenance dimension since we can integrate the characteristics of adaptation (e.g., type, timing, and reasons) that modify the program during its delivery.^45^

### 3.6 Maintenance

Studies discussed most points regarding the maintenance dimension (see Table 3). Behavioral or perceptual level assessment measures were used with participating parents and athletes (when necessary). The moment for assessment was divided into three different situations, such as before, during, and after the program,^17^, ^19^ as well as just before and after the program, or only after the end of the program. Additionally, 10 studies provided important information on suggestions for improvement in the programs, coming from parents, athletes, managers, and the researchers/deliverers themselves. Among the suggestions for improvement, ideas included (a) adaptations for a digital model; (b) improvements in the structure of materials such as videos and quizzes; (c) increased moments for parents to exchange experiences; and (d) changes regarding the clarity of instruction in some tasks.^11^, ^14^, ^15^, ^16^, ^25^, ^32^, ^35^, ^37^, ^42^, ^43^

The maintenance dimension is the only one in which the focal point is both the setting and individual level. According to Holtrop et al.,^23^ at the setting level, it is helpful to report whether the program is still ongoing post‐study and, if possible, adaptations were made. Again, similar to coach education programs,^30^ the current study showed limited data about sustainable programs for organizations. This means there was limited insight into whether programs would or could be continued after study completion (e.g., change in the organization's values to maintain the program in the future).^45^

However, a 4‐year evaluation using the RE‐AIM framework by Thrower et al.^25^ has provided valuable insights. For instance, to ensure program sustainability, a non‐profit fee may be necessary for parents who are interested in attending. In 2019, nearly half of the scheduled workshops were canceled (30 out of 72), demonstrating the program's adaptability. Despite variations in the delivery format over the 4 years, the average feedback scores for instructor evaluation, satisfaction, enjoyment, and transfer intention remained consistently high. Particularly, it may be useful for researchers to understand the “whys” of whether the sports organization decides to continue the program in their setting.^52^

At the individual level, there is a need to elucidate the long‐term outcomes after the program's conclusion.^23^ Regarding assessments of participant behavior, the studies included in the current review used specific assessment strategies, most often related to choices of theoretical or conceptual support and did so at least once (after completion of the program). As a result, this represented a diversity of measures, especially questionnaires used for assessments with either parents or athletes.^8^ Suggestions for improvement of evaluation in parent programs concern greater consideration for follow‐up assessments with participants, continuous contact during the program, and diagnostics with external parent support agents to verify long‐term behavior changes (e.g., coaches, athletes, or peers^8^, ^12^, ^23^, ^53^).

## 4 SUGGESTIONS FOR FUTURE PARENT EDUCATION PROGRAMS

Using the RE‐AIM approach, it is possible to identify and address multiple factors at different levels that impact parent education programs in sports. To ensure effective reach, it is essential to understand the characteristics of the parent population that a program seeks to benefit. This knowledge can help determine the most suitable content delivery strategy, such as using an online program for parents who work long hours. Additionally, it enables anticipating potential outreach challenges based on the target profile of participants. It is also important to identify the target number of parents who will benefit from the program to monitor those who drop out or fail to complete it. Recruitment can involve conducting surveys and direct outreach through emails, questionnaires, social media, and face‐to‐face interactions at the sports organization.^45^ Parents can be informed about the full program after participating in a short initial activity on the topic. This approach has been shown to be effective in generating more engagement, especially among people with higher incomes, education levels, and literacy in the program's subject matter.^54^

To optimize the efficacy of the program, practitioners must transcend mere participation rates. Implementation of evaluative mechanisms is imperative to gauge program effectiveness and longitudinally monitor participant outcomes. For instance, the incorporation of follow‐up assessments with parents and/or athletes who prematurely disengage from the program facilitates sustained engagement and furnishes essential support within subsequent program iterations. Furthermore, enhancing program monitoring and evaluation through soliciting feedback from stakeholders and program facilitators is paramount. Such feedback serves as a catalyst for continuous refinement, ensuring program pertinence, efficacy, and stakeholder engagement endure over time.

When designing adoption programs for sports organizations, it is essential to consider the characteristics of the organization, such as whether it is a federation, club, or sport academy. Cultural or financial barriers may prevent the program from being adopted by the institution. Research teams working with research organizations are crucial for successful program implementation. However, it may be beneficial to look at the example of Thrower et al.,^25^ who partnered with those interested in developing the program through a contractual knowledge exchange. This can help identify suitable staff to deliver the program, whether they need training, and whether there is a history of similar actions in the organization for parents. Additionally, sport organizations should evaluate the acceptability, burden, and relative advantage of parent education programs during and after implementation (see Goorevich et al.^55^).

To ensure that the program is implemented effectively, it is important to report on the cost and time measures. This involves providing details on how and when these resources were invested throughout the program (i.e., 1 week of staff training or buying a website license to host the program). These measures can be combined with information on the reach (i.e., the number of participants) and effectiveness (i.e., the proportion of participants who completed at least half of the program) to determine the minimum investment required per individual. Another important factor to consider is the documentation of any adaptations made to the original implementation protocol, which can help to assess necessary changes to the program for consistency and compatibility over time. For example, it would be beneficial to include feedback from sport managers, staff, and non‐completing participants as part of the evaluation program's workflow, in addition to feedback from parents, coaches, and athletes.

In the domain of Maintenance, practitioners wield the capacity to bolster the durability of parent education programs within sports, thereby augmenting their enduring impact and sustainability. This can be achieved through strategic interventions such as optimizing the structural integrity of educational materials by integrating multimedia components such as videos and quizzes. It is imperative that these materials are meticulously crafted to captivate audiences, facilitate seamless navigation, and proficiently impart salient concepts and skills to participating parents. Furthermore, fostering collaborative partnerships with external stakeholders, including coaches, athletes, or peers, serves to fortify the support network available to parents enrolled in the program, thereby fortifying its resilience and efficacy over the long term.

## 5 LIMITATIONS AND FUTURE RESEARCH DIRECTIONS

As this review focused on assessing studies of parent education programs using the RE‐AIM framework, we did not analyze the quality of the studies included based on factors such as study design, sampling procedures, data collection methods, and type of analysis used, among others. Recognizing the challenges of evaluating parent education programs,^8^ future research may consider using the RE‐AIM framework for planning, implementing, and evaluating parent education programs in sport. Specifically, using the five dimensions may provide an interactive guide for the program—although, considering which are assessed, adopting only a few dimensions of the framework is a possible way to plan and evaluate programs, as well.^23^ In addition, the field needs to develop evidence‐based programs based on the RE‐AIM framework in a diversity of sports environments and with multiple stakeholders looking for recommendations to refine the viability of this framework in sport.

Another constraint is that scholarly works commonly adopt a Western‐oriented stance toward parental education. Undoubtedly, Western notions of “quality parenting” make a substantial contribution. Nonetheless, it takes the risk of researching and dialoguing with the same community of members, all from the same cultural spectrum. In other words, it is prudent to recognize the likelihood that a wealth of insights from alternative contexts (such as Eastern perspectives on design and evaluate education programs to support parents in sports) remains untapped or undiscovered (see Dorsch et al.^56^).

## 6 PERSPECTIVE

Enhancing parental involvement in sport is a key consideration for the discipline of sport psychology. The current review provides a clear indication of the areas of sport parenting interventions that have been considered to date. However, it is clear that to enhance the quality of research that practitioners and organizations are drawing upon, there is a need for future program evaluations to increase the attention given to the reach, adoption, and implementation of programs. Potential strategies could be to apply RE‐AIM in different sports scenarios or to combine it with another evaluation framework. Additionally, practitioners and scholars can start using RE‐AIM in a pragmatic way by focusing on one dimension of the framework to address a specific issue. For example, they could use the reach dimension to improve parents' adherence to the programs.

## 7 CONCLUSION

In summary, this review advocates for the potential of drawing upon the RE‐AIM framework to facilitate planning and evaluation of parent education programs in sport—the study by Thrower et al.^25^ serves as a great foundation for further exploration in this area. However, it is also a useful framework for encouraging authors to consider the breadth of information and considerations that need to be included when developing and publishing interventions. Within parent education programs, it is clear authors are attending to the effectiveness of their programs and some elements of how they are being maintained. However, to enhance programs moving forwards and particularly the effectiveness of programs to encourage adoption by organizations, further specific consideration of the program's implementation would be useful, as well as the reach and adoption dimensions of the framework. Future researchers and practitioners may benefit from drawing on the RE‐AIM framework as an evidence‐based resource to increase the amount and quality of the information included in the design, implementation, and evaluation of parent education programs.

## FUNDING INFORMATION

This study was supported by the Coordenação de Aperfeiçoamento de Pessoal de Nível Superior ‐ Brazil (CAPES) under grant 001.

## CONFLICT OF INTEREST STATEMENT

No potential conflict of interest was reported by the author(s).

## Supporting information


Data S1.

## Data Availability

The data that support the findings of this study are available from the lead author upon reasonable request.

## Footnotes

* The article by Thrower et al.^25^ was first published online on December 07, 2022, at Loughborough's Research Repository and thus included in this study, although its final publication date is 2023.

^†^
Studies in which parents/families were one of several targeted groups but did not participate at any time in the program assessment process were excluded.

^‡^
We also have examples that focus only on families' participation^41^ and contacted just one specific sport organization for the program.^33^

^§^
Some programs allowed parents to manage their own time for the sessions/modules.^15^, ^18^, ^39^
