# Supplementary material for: An integrative review of parent education approaches in sport: Considerations for program planning and evaluation
Source: Scand J Med Sci Sports. 2024 Apr 6;34(4):e14620. doi: 10.1111/sms.14620 (PMC12810438; doi:10.1111/sms.14620)
Supplement: Supplementary file 1 — Data S1. [file SMS-34-e14620-s001.pdf]

Suplementary Document – RE-AIM Checklist

**Article Citation**			
**REACH**	**Reported (Yes/No)**	**Data**	**Comments**
**Method to identify the target population**			What was the selection process for the target population (e.g. all parents/family members selected for the current study were identified by their sport organization as meeting our selection criteria)
Description of the target population	NC		Description of the target population of parents/family members, as opposed to just the study sample (e.g. detailed demographic information)
Recruitment strategies	NC		How participants were recruited (e.g. we presented the study in front of a sports organization and emailed all eligible parents/family members)
**Inclusion criteria**			What criteria were used to determine who was eligible?
**Exclusion criteria**			What criteria made individuals ineligible?
**Sample size and participation rate**			Sample size / denominator
Denominator target population	NC		The number of parents/family members who were eligible and invited to participate
**Characteristics of participation and non-participation**			Why or what were the characteristics of the parents/family members who participated or not in the intervention? (why participated or not)
Use of qualitative methods to measure reach	NC		Reporting on non-quantitative aspects of reach

**EFFECTIVENESS**	**Design**	**Conditions**	
Methods' information will be grouped in a singular table			
	**Reported (Yes/No)**	**Effect size and gross results (when applicable)**	**Comments**
**Measures / results (in the shortest assessment)**			What was the main outcome measured to assess the effectiveness/effectiveness of the intervention and when was it assessed?
Results (on completion in the program)	NC		The size of the effect or the amount of change in parent/family outcomes (sometimes measured across the athlete) at the end of the program?
Assess unintended (negative) consequences and outcomes	NC		Did the study assess the potential for negative or unwanted outcomes? (challenges and barriers)
**Were the parents/family members who withdrew from the intervention evaluated or only those who were hired?**			Were participants' results analyzed even if they did not complete the intervention? Or alternatively, did the analysis relate only to those who successfully completed the intervention and follow-up measures?
Attribution procedures (please specify)	NC		Were procedures used to impute missing data to results?

**Attrition percentage (parents/family members completing the program)**			Did the study report on parents/family members who initiated the intervention but did not complete it, or provided follow-up responses? (eg, 6 parents/family members completed the study, out of 9 parents/family members who started initially)
Use of qualitative methods to measure efficacy/effectiveness	NC		Obtaining qualitative feedback from participants on the degree to which they felt the intervention was efficacious/effective

ADOPTION	Reported (Yes/No)	Data	Comments
**Description of the intervention location**			What were the specific details of where the intervention took place?
**Description of the team that delivered the intervention**			Specific characteristics of the team that conducted the intervention
**Method to identify the target delivery agent**			Were there any criteria used to select who delivered the intervention?
**Delivery agent expertise level**			Training or experience of the team that delivered/delivered the intervention?
**Inclusion/exclusion criteria**			There was some criteria for selecting which organizations participated in the study. How were they selected? Were criteria used to select who did and who did not deliver the intervention?
**Adoption rate (# participating configurations / total configurations)**			Number of participating organizations / number of contacted and eligible organizations
Characteristics of adoption / non-adoption	NC		What characteristics distinguish organizations that have or have not adopted the intervention program?
Use of qualitative methods to measure adoption	NC		Used qualitative methods to understand the process of adoption.

IMPLEMENTATION	Reported (Yes/No)	Data	Comments
Identification of theoretical support used	NC		Did the authors report a specific theory or principle that drove the intervention (eg, parental expertise)?
**Number of intervention contacts**			Total number of times contact was made with each participant (e.g., face-to-face; phone call; newsletters; emails, intervention sessions).
**Extension protocol delivered as intended**			Was information available to judge the extent to which the program was delivered as planned?
Participant participation/completion fees	NC		To what extent did participants attend/participate in all intervention sessions/meetings
**Cost measures**			What is the ongoing cost to implement the intervention? (example: time and money)
Pilot test	NC		Has the intervention pilot been tested in the organization?
Use of qualitative methods to measure implementation	NC		Used qualitative methods to understand the process of implementation.

MAINTENANCE	Reported (Yes/No)	Data	Comments

**Was individual behavior assessed at any time after completion of the intervention?**			Were the behavior of the parents/family members, their perceptions or the results through the athletes evaluated at a time after the conclusion of the intervention? (follow-up)
**At what level of intervention was the program in place?**			Was the intervention implemented at what contextual level (regional, national, international)?
**Has the program been modified? Specify**			Is there information about necessary changes to the program structure?
Use of qualitative methods to measure organizational level maintenance	NC		Used qualitative methods to understand the process of intervention sustainability at the organizational level

**TOTAL RE-AIM SCORE**	**20**		Quality range extends from low (0 – 6), to moderate (7 – 13) and high (14–20)
**Total Score Article**			

NC* - Non calculated as a score - further information and description throughout the results section
